# Dietary Histamine Impairs the Digestive Physiology Function and Muscle Quality of Hybrid Grouper (*Epinephelus fuscoguttatus*♀ × *Epinephelus lanceolatus*♂)

**DOI:** 10.3390/antiox12020502

**Published:** 2023-02-16

**Authors:** Yumeng Zhang, Hang Zhou, Yu Liu, Lulu Zhu, Jiongting Fan, Huajing Huang, Wen Jiang, Junming Deng, Beiping Tan

**Affiliations:** 1 College of Fisheries, Guangdong Ocean University, Zhanjiang 524088, China; 2 Aquatic Animals Precision Nutrition and High Efficiency Feed Engineering Research Centre of Guangdong Province, Zhanjiang 524088, China; 3 Key Laboratory of Aquatic, Livestock and Poultry Feed Science and Technology in South China, Ministry of Agriculture, Zhanjiang 524088, China

**Keywords:** histamine, hybrid grouper, growth performance, antioxidant response, digestive physiology function, muscle texture

## Abstract

An 8-week feeding experiment was conducted to investigate the effect of dietary histamine on growth performance, digestive physiology function and muscle quality in a hybrid grouper (*Epinephelus fuscoguttatus*♀ × *Epinephelus lanceolatus*♂). Seven isoproteic (50%) and isolipidic (11%) diets were prepared with various histamine inclusion levels of 0, 30, 60, 120, 240, 480 and 960 mg/kg in diets (actual contents were 72.33, 99.56, 138.60, 225.35, 404.12, 662.12 and 1245.38 mg/kg), respectively. Each diet was randomly assigned to triplicates of 30 juveniles (average body weight 14.78 g) per tank in a flow-through mariculture system. The increase in the dietary histamine level up to 1245.38 mg/kg made no significant difference on the growth rate and feed utilization of the grouper. However, the increased histamine content linearly decreased the activities of digestive enzymes, while no differences were observed in groups with low levels of histamine (≤404.12 mg/kg). Similarly, high levels of histamine (≥404.12 mg/kg) significantly damaged the gastric and intestinal mucosa, disrupted the intestinal tight junction structure, and raised the serum diamine oxidase activity and endotoxin level. Meanwhile, high doses of histamine (≥662.12 mg/kg) significantly reduced the activities of antioxidant enzymes, upregulated the relative expression of Kelch-like ECH-associated protein 1, and hardened and yellowed the dorsal muscle of grouper. These results showed that dietary histamine was detrimental to the digestive physiology function and muscle quality of the grouper, although it did compromise its growth performance.

## 1. Introduction

As the main protein source of aquafeed, fishmeal is widely used in the feed of carnivorous fish. Thus, the quality of fishmeal has attracted considerable attention in the aquaculture industry [1,2,3]. During the processing and storage of fishmeal, a hazardous level of histamine is easily produced by the microbial decarboxylation of free histidine present in fishmeal [4,5]. Thus, histamine is regarded as a good indication of the quality of fishmeal [6,7]. Meanwhile, histamine can cause the poisoning of animals, including fish [8]. Previous studies have shown that high levels of histamine in diets can reduce the growth rate and increase the mortality rate of chickens, as well as cause stomach erosion [9,10]. However, dietary histamine has different effects on the growth of various fish species. Dietary histamine inhibited the growth of American eel (*Anguilla rostrata*) [11], damaged the gastrointestinal tract structure of yellow catfish (*Pelteobagrus fulvidraco*) [12] and rainbow trout (*Oncorhynchus mykiss*) [13], and reduced the antioxidant capacity and immunity of American eel [14]. Conversely, dietary histamine had no negative impact [15,16] and even improved the growth performance of other fish species [12,17]. Therefore, the potential effects of dietary histamine on the growth and health of fish need to be further clarified.

The hybrid grouper (*Epinephelus fuscoguttatus*♀ × *E. polyphekadion*♂) has a bright future because of its fast growth and delicate flesh [18], and is widely farmed in China and Southeast Asia [19,20]. The hybrid grouper, as a typical carnivorous fish, has a high protein requirement (about 45–50%) in compound feed, with a high level of fishmeal [21]. In addition, the hybrid grouper is mostly farmed in high temperature and high humidity environments, and the diets are easily deteriorated to produce a large amount of histamine. However, the effects of dietary histamine on the growth performance and digestive physiology function of the hybrid grouper have been less reported. Thus, the present study was conducted to clarify the effects of dietary histamine on growth performance, digestive enzyme activities, the structure and function of the gastrointestinal tract, and the dorsal muscle texture and color, and thereby determine the tolerance level of the hybrid grouper to dietary histamine.

## 2. Materials and Methods

### 2.1. Experimental Feed

Fish meal, soy protein concentrate and soybean meal were used as the main protein sources, and soybean oil and soybean lecithin were used as the main lipid sources. Histamine dihydrochloride (purity ≥ 98%, Macklin Biochemical Co., Ltd., Shanghai, China) was selected as the source of histamine (Table 1). Our preliminary investigation showed that the histamine content in 130 commercial grouper diets ranged from 59 to 1120 mg/kg (Table S1). Seven isoproteic and isolipidic diets (named H0, H3, H6, H12, H24, H48 and H96) were prepared with histamine inclusion levels of 0, 30, 60, 120, 240, 480 and 960 mg/kg; the actual histamine contents were 72.33, 99.56, 138.60, 225.35, 404.12, 662.12 and 1245.38 mg/kg diets. All materials (expect for lipid sources) were crushed and passed through a sieve with a 60-mesh diameter, and then weighed according to the feed formula and mixed evenly. Soybean oil and soybean phospholipid were then added to the mixture and re-blended. Finally, approximately 25% distilled water was added to make a dough, and the dough was squeezed into 2.5 mm pellets by a double helix extrusion mechanism (F-26 type; South China University of Technology, Guangzhou, China). The feeds were placed in the shade to dry naturally for two days and then stored at −20 °C.

### 2.2. Fish and Experimental Conditions

Experimental fish were purchased from a commercial fish farm (Zhanjiang, China). A total of 630 healthy and uniform juveniles (initial body weight 14.78 ± 0.01 g) were randomly assigned into 7 groups with triplicates of 30 juveniles per tank (0.3 m^3^). Fish were fed twice daily at 8:00 and 17:00 for 8 weeks, and a siphon tube was used to remove feces from the tank daily to ensure the water quality in the flow-through mariculture system. During the experimental period, all tanks were kept under natural light conditions, with the water temperature at 26–30 °C and the dissolved oxygen level above 6 mg/L.

### 2.3. Sample Collection and Pre-Treatment

After the feeding trial, all fish were fasted for a day. The fish from each tank were weighed and counted to calculate the growth index, then anesthetized with eugenol solution. Four fish per tank were randomly selected, weighed or measured by body length, body weight, visceral mass weight and liver weight for the calculation of morphological indices.

Blood samples were removed from four fish per tank with a 1 mL syringe and placed at 4 °C for 12 h, and the serum was collected and stored at −80 °C. The stomach, foregut (one third of the intestinal segment near the stomach end) and hindgut (one third of the intestinal segment near the excretory opening) of six fish per tank were quickly put into liquid nitrogen for temporary storage and then stored at −80 °C for enzyme activity analyses. The hindguts of another four fish per tank were harvested and placed in an Eppendorf tube with RNA, which were later stored at 4 °C for a day and then put in a −80 °C refrigerator for an analysis of the relative mRNA expression.

In addition, the hindgut of four fish per tank were placed in 2.5% glutaraldehyde fixation solution and 4% paraformaldehyde solution, and the stomach was placed in 2.5% glutaraldehyde fixation solution for histological observation, respectively. The dorsal muscles were stripped and cut to the size of 3 cm × 3 cm × 1 cm for the analysis of textural characteristics and coloration.

### 2.4. Chemical Composition Analysis

The chemical composition of the experimental diets was determined with reference to the AOAC standard methods [22]. The moisture was dried at 105 °C to constant weight; crude protein was determined by an automatic Kjeldahl apparatus (Kjeltec^TM^ 8400; Foss, Hoganas, Sweden); crude lipid was extracted with petroleum ether by the Soxhlet method; and crude ash was incinerated at 550 °C for 6 h. The content of histamine in the diet was determined by an enzyme-linked immunosorbent assay (ELISA) kit (Shanghai Enzyme Linked Biotechnology Co., Ltd., Shanghai, China).

### 2.5. Biochemical Indexes Analyses

In total, a 0.1 g sample of stomach/foregut/hindgut and a ninefold volume ice-cold saline solution was mixed and homogenized using a homogenizer (T 25 digital ULTRA-TURRAX^®^, IKA, Staufen, Germany), and the supernatant was collected after centrifugation at 3500 rpm/min for 10 min at 4 °C. The total antioxidant capacity (TAC), catalase (CAT), and superoxide dismutase (SOD) activities, as well as the malondialdehyde (MDA) content in the serum and hindgut, and the activities of lipase, maltase and amylase in the foregut, were measured by commercial kits (Nanjing Jiancheng Bioengineering Institute, Nanjing, China) according to the kit’s instructions. The activity of pepsin in the stomach, the content of endotoxin and the activities of peroxidase (POD), glutathione peroxidase (GPx), glutathione reductases (GR) and diamine oxidase (DAO) in the serum and hindgut, and the activities of trypsin, Na^+^/K^+^-ATPase and Ca^2+^/Mg^2+^-ATPase in the foregut were measured using commercial kits (Shanghai Enzyme Linked Biotechnology Co., Ltd., Shanghai, China), according to the kit’s instructions.

### 2.6. Histological Observation

The hindgut was soaked in a 4% formaldehyde solution for one day and then transferred to 70% ethanol for preservation. The hindgut was dehydrated in different concentrations of ethanol (70%, 80%, 90%, 95%, 100%), immersed in xylene solution to make it transparent and then embedded in paraffin. It was cut into sections of 5–7 μm thickness, stained by hematoxylin and eosin (H&E) and sealed with neutral gum. The slides were observed in a fluorescent inverted microscope (Nikon Eclipse Ni-U; Nikon, Tokyo, Japan), and ten folds per section were randomly selected to measure the fold height, fold width and muscle thickness by image acquisition software (CellSens Standard 1.8, Pooher Optoelectronics (Shanghai) Technology Co., Ltd., Shanghai, China). The hindgut and stomach were placed in a 2.5% glutaraldehyde fixed solution for 24 h; the production of intestinal transmission electron microscopy (TEM) sections was performed with reference to Huang’s method [23]. The scanning electron microscopy (SEM) sections of the stomach were made with reference to Chen’s method [24].

### 2.7. Extraction of RNA and Real-Time Quantitative PCR Analysis

The total RNA from the hindgut was extracted using TransZol Up Plus RNA Kit (Beijing TransGen Biotech Co. Ltd., Beijing, China). The integrity of the total RNA was confirmed by 1% agarose gel electrophoresis, and the purity and concentration of the total RNA were evaluated spectrophotometrically (A260:280 nm). Then, the PrimeScript^TM^ RT eagent Kit (Takara, Tokyo, Japan) was used to reverse transcribe the RNA into cDNA according to the kit’s instructions. Based on the sequences in Gen Bank, gene-specific primers were designed using Primer 5 software (Table 2), and β-action was selected as the reference gene. The relative expression levels of the target genes were calculated using the 2^−ΔΔCT^ method [25]; H0 was the reference group.

### 2.8. Muscle Texture and Color Analysis

The textural characterization of the dorsal muscle was analyzed by the texture analyzer (TMS-PRO, Food Technology Corporation, Sterling, VA, USA). The color of the dorsal muscle was measured using a color colorimeter (Chroma Meter CR-400, Konica Minolta, Inc., Tokyo, Japan).

### 2.9. Calculations and Statistical Analysis

Formulas for growth performance and body index are as follows:

Weight gain (WG) = (*W*_f_ − *W*_i_)/*W*_i_;

Specific growth ratio (SGR, %/d) = 100 × (Ln *W*_f_ − Ln *W*_i_)/t;

Voluntary feed intake (VFI, mg/MBW/d) = *FI*/((*W*_i_ + *W*_f_)/2)/*t* × 1000;

Feed conversion ratio (FCR) = *FI*/(*W*_f_ − *W*_i_);

Protein efficiency ratio (PER) = (*W*_f_ − *W*_i_)/(*F* × *CP*);

Survival rate (SR, %) = 100 × (*N*_f_ − *N*_i_)/*N*_i_;

Hepatosomatic index (HSI, %) = 100 × *W*_l_/*W*_b_;

Viscerosomatic index (VSI, %) = 100 × *W*_v_/*W*_b_;

Condition factor (CF, g/cm^3^) = 100 × *W*_f_/*L*^3^;

Where *W*_f_ and *W*_i_ are the final body weight (g) and initial body weight (g); *t* is feeding days (d); *FI* is feed intake per fish (g); *CP* is crude protein content of feed (%); *N*_f_ and *N*_i_ are final fish number and initial fish number; and *W*_b_, *W*_v,_ *W*_l_ and *L* are body weight (g), viscera weight (g), liver weight (g) and body length (cm).

The data were analyzed by a one-way analysis of variance followed by Duncan’s multiple range tests. A significant difference was set at the level of *p* < 0.05. For detecting the potential linear or quadratic effects of the dietary histamine level, all data were also subjected to polynomial orthogonal contrast analysis. Statistical analysis was performed using SPSS 21.0 (SPSS Inc., Chicago, IL, USA) for Windows.

## 3. Results

### 3.1. Growth Performance

After the 8-week feeding trial, the SR of the grouper ranged from 90.00% to 97.78%, and no significant difference was found among the dietary groups (*p* > 0.05, Table 3). Similarly, no significant differences were observed in the WG, SGR, VFI, FCR, PER, HSI, VSI and CF of the grouper among the dietary groups (*p* > 0.05). However, the SR, PER and CF showed negative linear (*p* < 0.05) and quadratic (*p* < 0.05) trends in response to the dietary histamine level, while the HSI and VSI exhibited a negative linear trend in response to the dietary histamine level (*p* < 0.05).

### 3.2. Digestive Enzyme Activity

The dietary histamine level did not affect the foregut maltase and Ca^2+^/Mg^2+^-ATPase activities (*p* > 0.05, Table 4). The activities of pepsin, foregut trypsin, lipase, amylase, and Na^+^/K^+^-ATPase showed significantly negative linear (*p* < 0.01) and quadratic (*p* < 0.01) trends in response to the dietary histamine content; meanwhile, no significant differences were observed in the pepsin and lipase activities among the H0, H3, H6, and H12 groups, in the trypsin and amylase activities among the H0, H3, and H6 groups, and in the Na^+^/K^+^-ATPase activity among the H0, H3, H6, H12, and H24 groups (*p* > 0.05).

### 3.3. Intestinal Permeability

**Figure 1:**
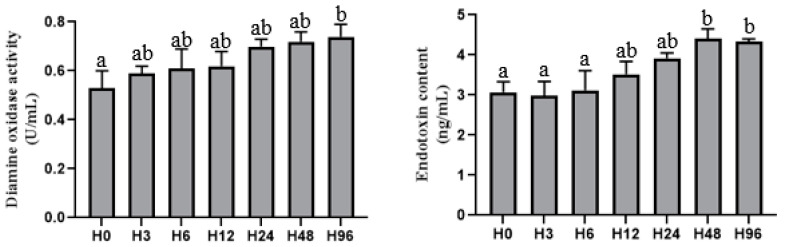
Effect of dietary histamine content on the intestinal permeability-related index in the serum of the hybrid grouper. Values are means ± SE of three replications. Different superscript letters in the bars indicate a significant difference among treatments by Tukey’s test (*p* < 0.05).

The serum DAO activity and endotoxin level increased with the rising dietary histamine content (Figure 1). Thus, the DAO activity was significantly higher in the H96 group compared to the H0 group (*p* < 0.05), and the endotoxin level was significantly higher in the H48 and H96 groups compared to the H0, H3 and H6 groups (*p* < 0.05).

### 3.4. Gastrointestinal Tract Structure

#### 3.4.1. SEM of Gastric Mucosa Cell

**Figure 2:**
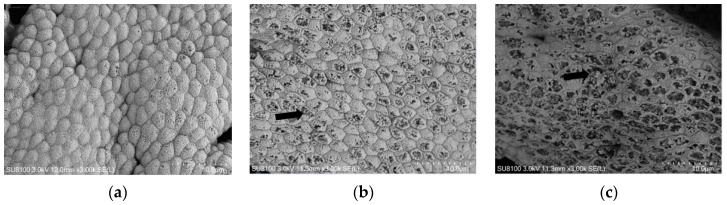
Scanning electron microscopy of gastric mucosa in the hybrid grouper fed with various dietary histamine levels. (**a**) indicates H0 group; (**b**) indicates H24 group; and (**c**) indicates H96 group. Arrows indicate cell damage. The cells of gastric mucosa cells in the H0 group were normal and tightly arranged; some cells in the H24 group were ruptured and the rest were relatively intact; most cells in the H96 group were severely damaged. The magnification was ×3.00 k, and the minimum scale (lower right) was 10.0 μm.

Damage to the gastric mucosal cells of the hybrid grouper was aggravated with the increasing dietary histamine content (Figure 2). In the H0 group, the gastric mucosa cells of the hybrid grouper were normal, with tightly arranged cells and clear borders; in the H24 group, some cells were ruptured and the rest were relatively intact; in the H96 group, most of the cells were severely damaged.

#### 3.4.2. Intestinal Morphology

**Figure 3:**
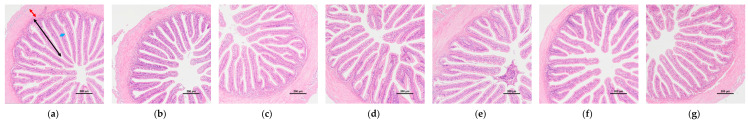
Effect of dietary histamine content on the hindgut histomorphology of the hybrid grouper. (**a**) indicates H0 group; (**b**) indicates H3 group; (**c**) indicates H6 group; (**d**) indicates H12 group; (**e**) indicates H24 group; (**f**) indicates H48 group; and (**g**) indicates H96 group. Black arrows: fold height (μm); blue arrows: fold width (μm); red arrows: muscular thickness (μm). The magnification was ×100, and the minimum scale (lower right) was 200 μm.

The dietary histamine content had no significant effect on the hindgut fold height and the muscular thickness of the grouper (*p* > 0.05, Table 5 and Figure 3). However, the hindgut fold width displayed the negative linear (*p* < 0.01) and quadratic (*p* < 0.05) trends in response to the dietary histamine level, which was significantly lower in the H96 group compared to the H0 group (*p* < 0.05).

#### 3.4.3. TEM of Intestinal Mucosal Cell

**Figure 4:**
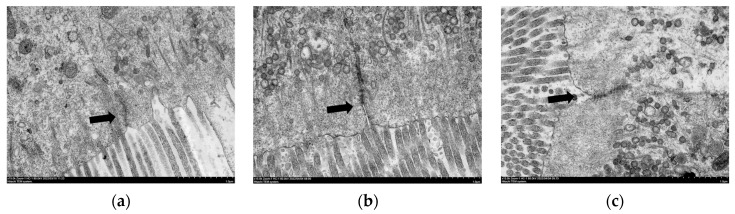
Transmission electron microscopy of intestinal mucosa in the hybrid grouper fed with various dietary histamine contents. (**a**) indicates H0 group; (**b**) indicates H24 group; and (**c**) indicates H96 group. Arrows indicate the junctions of the microvilli ends. The microvilli ends of intestinal mucosal cells in the H0 group were connected tightly between cells; in the H24 and H96 groups, gaps appeared between the microvilli ends of cells. The magnification was ×15.0 k, and the minimum scale (lower right) was 1.0 μm.

As shown in Figure 4, the linkage structure between the mucosal cells in the H0 group was tight and there were no gaps between the cells; meanwhile, in the H24 and H96 groups, gaps appeared at the microvilli end, indicating that the linkage structure between the mucosal cells was damaged.

### 3.5. Antioxidant Index

Serum SOD, CAT, POD, GPx, GR, and TAC activities exhibited significantly negative linear (*p* < 0.05) and quadratic (*p* < 0.05) trends in response to the dietary histamine level (Table 6); meanwhile, no significant differences were found in the SOD and GPx activities among the H0, H3, H6 and H12 groups, in the CAT and GR activities among the H0, H3, H6, H12 and H24 groups, in the POD activity among the H0, H3, H6, H12, H24 and H48 groups, and in the TAC activity among the H0 and H3 groups (*p* > 0.05). Conversely, the serum MDA level showed significantly positive linear (*p* < 0.001) and quadratic (*p* < 0.001) trends in response to the dietary histamine level, which was significantly higher in the H12, H24, H48 and H96 groups compared to the H0, H3 and H6 groups (*p* < 0.05).

The dietary histamine content had no significant effect on the activities of SOD and POD in the hindgut of the grouper (*p* > 0.05, Table 7), but the POD activity displayed negative linear (*p* < 0.05) and quadratic (*p* < 0.05) trends in response to the dietary histamine level. A negative linear trend was observed in the hindgut CAT activity (*p* < 0.05), and was significantly lower in the H96 group compared to the H0, H3, H6 and H12 groups (*p* < 0.05). In addition, the activities of GPx, GR and TAC in the hindgut exhibited significantly negative linear (*p* < 0.01) and quadratic (*p* < 0.05) trends in response to the dietary histamine level; meanwhile, no significant differences were found in the GPx and GR activities among the H0, H3, H6 and H12 group, or in the TAC activity among the H0, H3, H6, H12, H24 and H48 groups (*p* > 0.05). Conversely, the hindgut MDA level showed a significantly positive linear (*p* < 0.01) and quadratic (*p* < 0.01) trend in response to the dietary histamine level, which was significantly higher in the H96 group compared to the H0 group (*p* < 0.05).

### 3.6. Dorsal Muscle Texture and Color

The dietary histamine content did not affect the adhesiveness, springiness and resilience of the dorsal muscle (*p* > 0.05; Table 8). The hardness, cohesiveness, gumminess, and chewiness of the dorsal muscle exhibited significantly positive linear (*p* < 0.01) and quadratic (*p* < 0.01) trends in response to the dietary histamine level; meanwhile, no significant differences were observed in the hardness and chewiness among the H0, H3, H6, H12, H24 and H48 groups, or in the cohesiveness and gumminess among the H0, H3, H6, H12 and H24 groups (*p* < 0.05).

The L* and a* values of the dorsal muscle were not affected by the dietary treatments (*p* > 0.05; Table 9). However, the b* value showed significantly positive linear (*p* < 0.05) and quadratic (*p* < 0.05) trends in response to the dietary histamine content, which was significantly higher in the H48 and H96 groups compared to the H0 group (*p* < 0.05).

### 3.7. The Relative mRNA Expression of Tight Junction Proteins and Oxidative Stress-Related Factors

**Figure 5:**
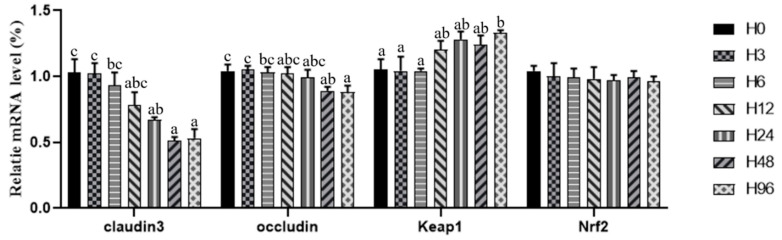
Effect of dietary histamine content on the relative mRNA expression of tight junction proteins and oxidative stress-related factors in the hindgut of the hybrid grouper. Keap1, Kelch-like ECH-associated protein 1; Nrf2, nuclear factor erythroid 2-related factor 2. Bars represent means ± SE of three replications. Different superscript letters in the bars indicate a significant difference among treatments by Tukey’s test (*p* < 0.05).

There was no significant difference in the relative expression level of nuclear factor erythroid 2-related factor 2 (Nrf2) among the dietary groups (*p* > 0.05; Figure 5). The relative expression levels of intestinal claudin3 and occludin gradually decreased with the increase in dietary histamine content; meanwhile, no significant differences were observed in the relative expression level of claudin3 among the H0, H3, H6 and H12 groups, or in the relative expression level of occludin among the H0, H3, H6, H12 and H24 groups (*p* < 0.05). Conversely, the relative expression level of Kelch-like ECH-associated protein 1 (Keap1) increased with the rising dietary histamine content, which was significantly higher in the H96 groups compared to the H0 group (*p* < 0.05).

## 4. Discussion

Dietary histamine is a toxin to some fish species [26,27] and has a negative impact on the growth of fish [15,16,28,29,30]. However, the effects of dietary histamine on the growth of fish vary with different fish species. It has been shown that a low level of dietary histamine does not affect the growth performance of yellow catfish [12,31], American eel [28] and rainbow trout [13], and even has a beneficial effect on the growth of yellow catfish [12] and Atlantic salmon (*Salmo salar* L.) [17]. In this study, the growth performance of the hybrid grouper was not affected by the dietary histamine content (72–1245 mg/kg), which is consistent with the results of previous studies with orange-spotted grouper (*Epinephelus coioides,* 158.7–2158.7 mg/kg) [3], yellow catfish (100–1000 mg/kg) [31] and American eel (67–414 mg/kg) [28]. Meanwhile, the SR and PER exhibited negative linear and quadratic trends in response to the dietary histamine level, suggesting that dietary histamine has a negative effect on the growth and health of the hybrid grouper. Liu et al. found that up to 2000 mg/kg of dietary histamine did not result in a remarkable reduction in growth, whereas 2500 mg/kg or more of dietary histamine could cause significant negative effects on the growth and health of the orange-spotted grouper [3]. Similar results were found in Atlantic Halibut (*Hippoglossus*) [32] and Atlantic Salmon (*Salmo Salar*) [17], where a low dose of dietary histamine had no significant effect on fish growth, but high levels of histamine (690 and 1742 mg/kg) reduced SGR significantly. Additionally, the growth performance of orange-spotted grouper was not statistically different among the dietary treatments at the initial feeding period (0–28 days), whereas dietary histamine suppressed the growth performance during the whole feeding period (0–56 days) [3]. Thus, the constant growth performance in this study may be attributed to the relative low content of dietary histamine and the relatively short-term feeding trial.

The gastrointestinal tract comprises the important digestive organs of fish and the main sites of digestion and absorption. The activities of digestive and absorptive enzymes reflect the digestive and absorptive capacity of fish [33], which is closely related to the growth and health of fish. Histamine regulates the secretion of gastric acid, and the intake of a certain amount of histamine by animals can promote gastric acid secretion, thereby affecting digestive enzyme activity [34]. Meanwhile, histamine intake can damage the structure and function of the fish intestine, and also inhibit digestive enzyme activities [14]. In this study, high doses of dietary histamine (≥225 mg/kg) decreased the digestive enzyme (pepsin, trypsin, lipase and amylase) activities of the hybrid grouper, which was similar to the results found with Chinese mitten crab [26] and American eel [14]. Thus, dietary histamine may disrupt intestinal structure and function, and thereby depress the digestive enzyme activities of the hybrid grouper.

The normal development of the intestinal tract is associated with the health of fish [35]. The surface area of intestinal absorption is related to the height of mucosal folds, and the muscularis is related to the abilities of intestinal peristalsis [23,36]. In this study, the width of the intestinal folds decreased linearly with the increased dietary histamine content, indicating that dietary histamine impaired the structure of the intestine. It is generally believed that dietary histamine exerts its pathological effects through histamine receptors in the gastric and intestinal mucosa [37], and thereby affects the structure and function of the gastrointestinal tract of the hybrid grouper. Previous studies showed that 10,000 mg/kg of histamine in the diet caused the epithelial exfoliation and the atrophy of lamina propria in the stomach mucosa of rainbow trout [16], and that 103.5 mg/kg of histamine damaged the gastric mucosa of yellow catfish [12]. In this study, SEM of gastric mucosa showed that some gastric mucosal cells of the hybrid grouper were destroyed by the dietary histamine content up to 404 mg/kg. These results indicated that the tolerance to histamine of fish vary with different fish species.

The intestinal barrier prevents the invasion of toxins, antigens and pathogens [38]. An intact intestinal cell structure and intercellular junction structure are associated with intestinal health in fish. The intestinal mucosa allows nutrients to enter the body while blocking pathogens [39]. Serum DAO activity and endotoxin level can, to some extent, reflect the degree of damage to the intestinal mucosa. Under normal conditions, DAO is mainly distributed inside the cells of the intestinal villi [40], and endotoxin is distributed in the intestinal lumen of the organism [41]. After damage to the intestinal mucosa, DAO and endotoxin enter the blood circulation through the intestinal mucosa, causing an increase in DAO activity [39] and endotoxin level [42]. In this study, serum DAO activity and endotoxin level increased with the increase in the dietary histamine content and damaged the connection structure between intestinal mucosal cells when the dietary histamine level exceeded 404 mg/kg. In addition, tight junction proteins (such as claudins and occludin) are a major part of the mechanical barrier of intestinal mucosa [43], which control the paracellular space between intestinal epithelial cells and prevent the spread of bacteria and antigens. In this study, the relative expressions of claudin 3 and occludin were markedly decreased by dietary histamine up to 404 mg/kg, indicating that a high dose of histamine increased the intestinal permeability and impaired the intercellular junctional structures of intestinal mucosa, leading to the entry of toxic substances into the bloodstream.

The antioxidant capacity of fish is mainly reflected by antioxidant enzymes, which protect the body from damage by removing the excessive accumulation of reactive oxygen species (ROS) [44]. Oxidative stress is a state of imbalance between oxidation and antioxidation that can have harmful effects on cellular organelles [45]. TAC is a comprehensive indicator of the reaction antioxidant capacity [46,47]. MDA is a main product of lipid peroxidation [48] and a sign of mucosal damage by ROS [49]. SOD, CAT, POD, GPx and GR are the main antioxidant enzymes in fish [50,51,52,53], which maintain the balance of oxidation and antioxidation [54]. High doses of dietary histamine increased the intestinal MDA level but decreased the intestinal TAC, SOD and CAT activities in American eel [14]. In this study, dietary histamine levels above 662 mg/kg decreased the antioxidant enzymes activities in the intestine of the hybrid grouper, but increased the MDA content. Nrf2-Keap1 is an important pathway that regulates oxidative stress [55,56], and Nrf2 is suppressed through Keap1-controlled ubiquitination-proteasomal degradation [57,58]. In this study, a high histamine level (1245 mg/kg) up-regulated the expression of Keap1 and thereby caused oxidative stress; there was an inability to scavenge oxygen radicals, which, in turn, led to the damage of the intestinal mucosa and intestinal epithelium.

Muscle texture can respond to the softness and elasticity of meat, which is an important indicator of meat consumption [59]; it not only affects the appearance of aquatic products, but also affects the taste [60]. Hardness reflects the internal binding force of meat to maintain its shape; cohesiveness reflects the magnitude of the intercellular binding force; and springiness and resilience reflect the biological resilience of fish [61]. In this study, the hardness, cohesiveness, gumminess and chewiness of the hybrid grouper were markedly increased when the dietary histamine level exceeded 662 mg/kg, indicating that high levels of histamine harden the muscle texture of the dorsal muscle and affect the taste of the hybrid grouper. In addition, the color of the meat is an indication of the quality of meat and an important factor affecting consumption [59,62]. The oxidation of muscle tissue lipids causes the meat to turn brown [63]. In this study, the b* value was markedly increased when the dietary histamine level reached 662 mg/kg. This may be due to the oxidation of the dorsal muscle, caused by a high dose of histamine, leading to the yellowing of the muscle color of the grouper.

In this study, although a high dose of dietary histamine depressed digestive enzyme activity, intestinal morphology and antioxidant capacity, resulting in a negative impact on intestinal health, it had no detrimental effect on the growth performance of the hybrid grouper. Similar situations were also observed in the research on yellow catfish [31], American eel [28] and rainbow trout [13]. The above phenomenon may be related to the short trial time, meaning that the damage to the organs was not yet visible in the organism. Furthermore, dietary 2500 mg/kg of histamine supplementation had no negative effect on the growth of the grouper in 28 days, whereas the SGR was significantly reduced in 56 days [3]. The above phenomenon can also be caused by the low content of histamine in the diet; its toxic effect was not sufficient to affect the growth of the hybrid grouper. Similar to our hypothesis, stating that dietary histamine levels above 1742 mg significantly reduce the SGR of Atlantic salmon, a dietary low dose of histamine had no significant effect [17]. Additionally, a dietary high dose of histamine (517 mg/kg) supplementation had a negative effect on the immune capacity of American eels [11]. Therefore, a dietary high dose of histamine supplementation may also reduce the immunity of the hybrid grouper, and future related studies are warranted.

## 5. Conclusions

Under this experimental condition, a dietary histamine content of no more than 1245 mg/kg did not negatively affect the growth performance of the hybrid grouper. However, the dietary histamine content up to 404 mg/kg impaired the structure and function of the gastrointestinal tract, inhibited the intestinal digestive enzyme activities and antioxidant response, and resulted in muscle sclerosis and the yellowing of the hybrid grouper.

## Figures and Tables

**Table 1:** Ingredients and chemical composition of the experimental diets (% dry matter).

Ingredients (%)	Diets						
	H0	H3	H6	H12	H24	H48	H96
Fish meal	45.00	45.00	45.00	45.00	45.00	45.00	45.00
Soy protein concentrate	21.00	21.00	21.00	21.00	21.00	21.00	21.00
Soybean meal	6.00	6.00	6.00	6.00	6.00	6.00	6.00
Wheat flour	17.91	17.91	17.91	17.91	17.91	17.91	17.91
Soybean oil	5.30	5.30	5.30	5.30	5.30	5.30	5.30
Soybean lecithin	2.00	2.00	2.00	2.00	2.00	2.00	2.00
Ca(H_2_PO_4_)_2_	1.20	1.20	1.20	1.20	1.20	1.20	1.20
Choline chloride	0.40	0.40	0.40	0.40	0.40	0.40	0.40
Vitamin C	0.03	0.03	0.03	0.03	0.03	0.03	0.03
Vitamin and mineral premixes ^1^	1.00	1.00	1.00	1.00	1.00	1.00	1.00
Histamine dihydrochloride (mg/kg) ^2^	0.00	50.70	101.39	202.78	405.56	811.13	1622.25
Cellulose microcrystalline (mg/kg)	1622.25	1571.55	1520.86	1419.47	1216.69	811.12	0.00
Proximate composition							
Dry matter (DM, %)	92.34	92.28	92.30	92.30	92.35	92.40	92.36
Crude protein (% DM)	49.50	50.20	48.97	49.18	49.23	50.11	49.79
Crude lipid (% DM)	11.27	11.30	11.28	11.28	11.28	11.27	11.26
Histamine (mg/kg DM)	72.33	99.56	138.60	225.35	404.12	662.12	1245.38

^1^ Supplied by Beijing Enhalor Biotechnology Co., Ltd., Beijing, China. ^2^ Supplied by Macklin Biochemical Co., Ltd., Shanghai, China; purity ≥ 98%.

**Table 2:** The forward and reverse primers used for real-time quantitative PCR analysis and accession numbers of gene sequences (GenBank).

Target Gene	Primer Sequence	GenBank Accession No.
claudin3	F-AGCCTTCATCGGCAGCAA R-GGATGCCTCGTCGTCAATG	EU714179.1
occludin	F-GGAGGAGAAACAGGGAATGAACT R-TCTGCTACAGCCTGGTATTTGG	KF861990.1
Keap1 ^1^	F-TCCACAAACCCACCAAAGTAA R-TCCACCAACAGCGTAGAAAAG	XM_018665037.1
Nrf2 ^2^	F-TATGGAGATGGGTCCTTTGGTG R-GCTTCTTTTCCTGCGTCTGTTG	KU892416.1
β-Actin	F-GGCTACTCCTTCACCACCACA R-TCTGGGCAACGGAACCTCT	AY510710.2

^1^ Keap1, kelch-like ECH-associated protein 1. ^2^ Nrf2, nuclear factor erythroid 2-related factor 2.

**Table 3:** Effects of dietary histamine content on growth performance, feed utilization and body index of hybrid grouper.

	Diets							*p*-Value	
	H0	H3	H6	H12	H24	H48	H96	Linear	Quadratic
Initial body weight (g)	14.77 ± 0.01	14.80 ± 0.01	14.76 ± 0.01	14.81 ± 0.00	14.76 ± 0.05	14.76 ± 0.01	14.77 ± 0.01	0.486	0.581
Final body weight (g)	116.39 ± 0.25	117.19 ± 1.49	115.01 ± 1.84	114.62 ± 1.03	114.76 ± 1.53	114.18 ± 0.46	113.57 ± 0.70	0.080	0.159
Weight gain	6.88 ± 0.03	6.81 ± 0.02	6.79 ± 0.13	6.74 ± 0.07	6.78 ± 0.09	6.73 ± 0.03	6.69 ± 0.05	0.128	0.285
Specific growth ratio (%/d)	3.62 ± 0.01	3.60 ± 0.00	3.60 ± 0.03	3.59 ± 0.01	3.60 ± 0.02	3.59 ± 0.01	3.58 ± 0.01	0.131	0.291
Voluntary feed intake (mg/MBW/d)	23.62 ± 0.50	23.26 ± 0.23	23.47 ± 0.42	23.31 ± 0.46	23.86 ± 0.15	23.61 ± 0.09	23.73 ± 0.39	0.414	0.634
Feed conversion ratio	0.83 ± 0.01	0.87 ± 0.01	0.87 ± 0.04	0.89 ± 0.02	0.88 ± 0.03	0.89 ± 0.02	0.90 ± 0.02	0.059	0.110
Protein efficiency ratio	2.44 ± 0.03	2.38 ± 0.04	2.40 ± 0.07	2.37 ± 0.03	2.31 ± 0.03	2.32 ± 0.07	2.29 ± 0.02	0.021	0.041
Survival rate (%)	96.67 ± 0.00	97.78 ± 1.11	96.67 ± 3.33	93.33 ± 1.93	91.11 ± 2.94	90.00 ± 3.33	90.00 ± 3.33	0.007	0.003
Hepatosomatic index (%)	4.55 ± 0.12	4.46 ± 0.29	4.20 ± 0.22	4.09 ± 0.18	4.02 ± 0.29	3.94 ± 0.22	3.87 ± 0.25	0.043	0.056
Viscerosomatic index (%)	12.79 ± 0.35	12.51 ± 0.61	12.16 ± 0.32	11.97 ± 0.46	11.86 ± 0.31	11.67 ± 0.42	11.50 ± 0.49	0.041	0.061
Condition factor (g/cm^3^)	3.01 ± 0.09	2.86 ± 0.12	2.82 ± 0.08	2.87 ± 0.10	2.74 ± 0.07	2.73 ± 0.05	2.73 ± 0.03	0.023	0.011

Values are means ± standard error (SE) of three replications. Different superscript letters in each row show significant differences among treatments by Tukey’s test (*p* < 0.05).

**Table 4:** Effect of dietary histamine content on the digestive enzyme activity in the gastrointestinal tract of the hybrid grouper.

	Diets							*p*-Value	
	H0	H3	H6	H12	H24	H48	H96	Linear	Quadratic
*Stomach*									
Pepsin (U/mg protein)	56.05 ± 2.82 ^bc^	58.39 ± 1.36 ^c^	51.32 ± 4.41 ^abc^	49.60 ± 3.32 ^abc^	45.57 ± 3.18 ^ab^	44.56 ± 3.72 ^ab^	42.57 ± 1.70 ^a^	0.009	0.008
*Foregut*									
Trypsin (U/μg protein)	0.25 ± 0.02 ^c^	0.25 ± 0.02 ^c^	0.22 ± 0.01 ^bc^	0.19 ± 0.01 ^ab^	0.19 ± 0.01 ^ab^	0.17 ± 0.01 ^a^	0.18 ± 0.01 ^ab^	0.002	<0.001
Lipase (U/g protein)	1.65 ± 0.04 ^c^	1.68 ± 0.06 ^c^	1.49 ± 0.06 ^abc^	1.54 ± 0.15 ^bc^	1.37 ± 0.07 ^ab^	1.26 ± 0.03 ^a^	1.27 ± 0.09 ^a^	<0.001	<0.001
Amylase (U/mg protein)	0.38 ± 0.00 ^c^	0.33 ± 0.02 ^bc^	0.31 ± 0.02 ^abc^	0.26 ± 0.02 ^ab^	0.29 ± 0.00 ^ab^	0.25 ± 0.03 ^ab^	0.25 ± 0.05 ^a^	0.009	0.005
Maltase (U/mg protein)	13.93 ± 1.80	13.30 ± 0.56	12.88 ± 0.52	12.58 ± 1.49	11.49 ± 1.56	10.83 ± 0.74	10.72 ± 1.51	0.077	0.112
Na^+^/K^+^-ATPase (U/mg protein)	1.64 ± 0.12 ^c^	1.63 ± 0.09 ^c^	1.48 ± 0.06 ^bc^	1.54 ± 0.08 ^bc^	1.46 ± 0.09 ^bc^	1.31 ± 0.03 ^ab^	1.14 ± 0.02 ^a^	<0.001	<0.001
Ca^2+^/Mg^2+^-ATPase (U/mg protein)	5.71 ± 0.49	5.73 ± 0.87	5.63 ± 0.43	5.52 ± 0.08	5.53 ± 0.74	5.12 ± 0.78	4.96 ± 0.07	0.213	0.450

Values are means ± standard error (SE) of three replications. Different superscript letters in each row show significant differences among treatments by Tukey’s test (*p* < 0.05).

**Table 5:** Effect of dietary histamine content on the intestinal histomorphology of the hybrid grouper.

	Diets							*p*-Value	
	H0	H3	H6	H12	H24	H48	H96	Linear	Quadratic
Fold height (μm)	643.16 ± 19.54	646.77 ± 17.25	647.34 ± 12.29	645.80 ± 19.92	637.39 ± 12.97	627.73 ± 7.95	617.98 ± 15.18	0.167	0.382
Fold width (μm)	82.28 ± 2.41 ^b^	79.04 ± 3.21 ^ab^	80.85 ± 3.14 ^ab^	80.48 ± 2.77 ^ab^	76.38 ± 3.16 ^ab^	73.16 ± 3.05 ^ab^	69.51 ± 2.63 ^a^	0.008	0.025
Muscular thickness (μm)	147.27 ± 7.00	144.93 ± 9.01	145.42 ± 8.22	154.83 ± 6.98	155.31 ± 6.42	138.20 ± 6.71	142.61 ± 10.33	0.531	0.823

Values are means ± standard error (SE) of three replications. Different superscript letters in each row show significant differences among treatments by Tukey’s test (*p* < 0.05).

**Table 6:** Effect of dietary histamine content on the antioxidant-related index in the serum of the hybrid grouper.

	Diets							*p*-Value	
	H0	H3	H6	H12	H24	H48	H96	Linear	Quadratic
SOD (U/mL)	62.85 ± 2.26 ^c^	61.64 ± 1.38 ^bc^	56.76 ± 1.81 ^abc^	56.70 ± 3.38 ^abc^	54.90 ± 1.50 ^ab^	55.35 ± 2.13 ^a^	52.83 ± 1.65 ^a^	0.003	0.002
CAT (U/mL)	9.71 ± 1.36 ^b^	8.75 ± 1.19 ^ab^	7.77 ± 0.37 ^ab^	7.23 ± 0.52 ^ab^	7.15 ± 0.79 ^ab^	6.72 ± 1.04 ^a^	6.10 ± 0.59 ^a^	0.009	0.017
POD (U/mL)	0.34 ± 0.01 ^b^	0.32 ± 0.02 ^ab^	0.30 ± 0.03 ^ab^	0.29 ± 0.01 ^ab^	0.30 ± 0.01 ^ab^	0.28 ± 0.02 ^ab^	0.26 ± 0.02 ^a^	0.004	0.014
GPx (U/mL)	0.17 ± 0.01 ^b^	0.15 ± 0.01 ^ab^	0.15 ± 0.01 ^ab^	0.15 ± 0.01 ^ab^	0.14 ± 0.00 ^a^	0.13 ± 0.00 ^a^	0.13 ± 0.01 ^a^	0.012	0.005
GR (U/mL)	0.15 ± 0.01 ^b^	0.15 ± 0.01 ^b^	0.13 ± 0.01 ^b^	0.13 ± 0.01 ^b^	0.12 ± 0.00 ^ab^	0.10 ± 0.00 ^a^	0.10 ± 0.01 ^a^	<0.001	<0.001
TAC (nmol/L)	1.83 ± 0.05 ^c^	1.72 ± 0.05 ^bc^	1.62 ± 0.03 ^ab^	1.62 ± 0.00 ^ab^	1.59 ± 0.04 ^ab^	1.55 ± 0.06 ^a^	1.53 ± 0.07 ^a^	0.002	0.001
MDA (nmol/L)	3.30 ± 0.43 ^a^	3.08 ± 0.24 ^a^	3.27 ± 0.22 ^a^	4.95 ± 0.44 ^b^	6.03 ± 0.71 ^b^	6.48 ± 0.47 ^b^	6.43 ± 0.58 ^b^	<0.001	<0.001

Values are means ± standard error (SE) of three replications. Different superscript letters in each row show significant differences among treatments by Tukey’s test (*p* < 0.05).

**Table 7:** Effect of dietary histamine content on the antioxidant-related index in the hindgut of the hybrid grouper.

	Diets							*p*-Value	
	H0	H3	H6	H12	H24	H48	H96	Linear	Quadratic
SOD (U/μg protein)	0.60 ± 0.01	0.58 ± 0.02	0.59 ± 0.02	0.56 ± 0.02	0.62 ± 0.02	0.61 ± 0.01	0.60 ± 0.03	0.284	0.315
CAT (U/mg protein)	17.46 ± 0.91 ^b^	17.51 ± 0.73 ^b^	16.82 ± 1.84 ^b^	16.85 ± 3.11 ^b^	15.85 ± 1.07 ^ab^	16.61 ± 0.85 ^ab^	13.21 ± 0.50 ^a^	0.047	0.139
POD (U/g protein)	27.13 ± 3.02	27.08 ± 0.57	25.57 ± 2.39	23.98 ± 3.43	22.16 ± 2.29	20.05 ± 3.16	18.69 ± 1.13	0.012	0.021
GPx (U/mg protein)	15.27 ± 1.31 ^c^	16.08 ± 0.46 ^c^	13.46 ± 0.57 ^bc^	12.79 ± 0.97 ^abc^	11.34 ± 0.84 ^ab^	9.57 ± 1.22 ^a^	9.64 ± 1.65 ^a^	0.001	<0.001
GR (U/mg protein)	2.49 ± 0.21 ^c^	2.62 ± 0.28 ^c^	2.31 ± 0.09 ^bc^	2.22 ± 0.15 ^bc^	1.77 ± 0.11 ^ab^	1.52 ± 0.21 ^a^	1.67 ± 0.16 ^a^	0.003	<0.001
TAC (mmol/g protein)	15.00 ± 2.64 ^b^	13.66 ± 1.46 ^ab^	13.07 ± 1.79 ^ab^	12.97 ± 1.14 ^ab^	10.57 ± 0.98 ^ab^	10.32 ± 2.07 ^ab^	8.75 ± 1.29 ^a^	0.007	0.014
MDA (nmol/g protein)	22.87 ± 5.92 ^a^	26.77 ± 5.08 ^ab^	27.63 ± 3.34 ^ab^	28.26 ± 2.36 ^ab^	29.00 ± 2.61 ^ab^	34.61 ± 6.42 ^ab^	39.31 ± 2.50 ^b^	0.001	0.004

Values are means ± standard error (SE) of three replications. Different superscript letters in each row show significant differences among treatments by Tukey’s test (*p* < 0.05).

**Table 8:** Effect of dietary histamine content on the dorsal muscle texture of the hybrid grouper.

	Diets							*p*-Value	
	H0	H3	H6	H12	H24	H48	H96	Linear	Quadratic
Hardness (kg)	0.95 ± 0.07 ^a^	0.96 ± 0.08 ^a^	1.06 ± 0.05 ^ab^	1.08 ± 0.09 ^ab^	1.16 ± 0.08 ^ab^	1.15 ± 0.09 ^ab^	1.25 ± 0.08 ^b^	0.003	0.008
Adhesiveness (g.sec)	−11.29 ± 2.74	−9.13 ± 1.22	−10.33 ± 1.91	−8.35 ± 1.00	−11.03 ± 2.14	−10.97 ± 2.29	−10.50 ± 2.06	0.729	0.922
Springiness	0.23 ± 0.02	0.26 ± 0.06	0.22 ± 0.02	0.28 ± 0.04	0.23 ± 0.03	0.23 ± 0.04	0.24 ± 0.05	0.817	0.961
Cohesiveness	0.10 ± 0.00 ^a^	0.09 ± 0.00 ^a^	0.10 ± 0.01 ^a^	0.12 ± 0.00 ^ab^	0.11 ± 0.01 ^ab^	0.13 ± 0.01 ^bc^	0.15 ± 0.01 ^c^	<0.001	<0.001
Gumminess	85.85 ± 13.39 ^a^	86.86 ± 7.46 ^a^	110.87 ± 13.44 ^ab^	115.02 ± 13.15 ^ab^	122.43 ± 3.75 ^ab^	118.84 ± 11.89 ^b^	113.67 ± 23.77 ^b^	0.003	0.005
Chewiness	20.75 ± 0.75 ^a^	19.94 ± 3.04 ^a^	27.83 ± 4.67 ^ab^	38.18 ± 5.83 ^ab^	36.23 ± 7.67 ^ab^	39.63 ± 8.51 ^ab^	42.88 ± 7.39 ^b^	0.008	0.008
Resilience	0.04 ± 0.00	0.04 ± 0.00	0.04 ± 0.00	0.04 ± 0.00	0.04 ± 0.00	0.04 ± 0.00	0.05 ± 0.01	0.123	0.262

Values are means ± standard error (SE) of three replications. Different superscript letters in each row show significant differences among treatments by Tukey’s test (*p* < 0.05).

**Table 9:** Effect of dietary histamine content on the color of the dorsal muscle of the hybrid grouper.

	Diets							*p*-value	
	H0	H3	H6	H12	H24	H48	H96	Linear	Quadratic
L value ^1^	59.52 ± 1.40	59.76 ± 1.43	59.71 ± 0.94	60.08 ± 0.84	60.10 ± 0.70	60.19 ± 1.73	59.34 ± 1.49	0.906	0.832
a value ^2^	−3.94 ± 0.13	−3.63 ± 0.32	−3.19 ± 0.46	−3.75 ± 0.33	−3.87 ± 0.24	−3.92 ± 0.12	−3.72 ± 0.31	0.710	0.840
b value ^3^	6.43 ± 0.29 ^a^	7.54 ± 0.70 ^ab^	7.59 ± 0.75 ^ab^	6.93 ± 0.36 ^ab^	7.77 ± 0.39 ^ab^	8.42 ± 0.28 ^b^	8.48 ± 0.28 ^b^	0.010	0.021

^1^ If the value is positive, the sample is brighter than the standard version; if it is negative, it is darker. ^2^ If the value is positive, the sample is redder than the standard version; if it is negative, it is greener. ^3^ If the value is positive, the sample is more yellow than the standard version; if it is negative, it is bluer. Values are means ± standard error (SE) of three replications. Different superscript letters in each row show significant differences among treatments by Tukey’s test (*p* < 0.05).

## Data Availability

Data are contained within the article and supplementary materials.

## Supplementary Materials

The following are available online at https://www.mdpi.com/article/10.3390/antiox12020502/s1, Table S1: Histamine levels in partially commercial feeds.
