# Supplementary material for: Dietary Histamine Impairs the Digestive Physiology Function and Muscle Quality of Hybrid Grouper (Epinephelus fuscoguttatus♀ × Epinephelus lanceolatus♂)
Source: Antioxidants (Basel). 2023 Feb 16;12(2):502. doi: 10.3390/antiox12020502 (PMC9952090; doi:10.3390/antiox12020502)
Supplement: Supplementary file 1 [file antioxidants-12-00502-s001.zip › antioxidants-2093163-supplementary.pdf]

Table S1. Histamine levels in partially commercial feeds.

Manufacturers	Feed Name	Histamine (mg/kg)
Aqua Master Enterprise Co.	Grouper compound feed EP504 grouper 4#	352
Aqua Master Enterprise Co.	Grouper compound feed EP504 grouper 5#	453
Seaford Aquatic Development Co.	Seawater fish expanded compound feed grouper feed	462
Seaford Aquatic Development Co.	Seawater fish expanded compound feed grouper feed	195
Shang Shang Biotechnology Co.	Grouper compound feed No. 3-4	293
Shang Shang Biotechnology Co.	Grouper compound feed No. 5-7	98
Yuejia Feed Co.	Grouper feed 8925	273
Hainan Haiyi Aquatic Feed Co.	Grouper feed 8925	335
Hainan Haiyi Aquatic Feed Co.	Grouper feed 8925	230
Hainan Haiyi Aquatic Feed Co.	Grouper feed 8925	203
Hainan Haiyi Aquatic Feed Co.	Grouper feed 8925	233
Hainan Haiyi Aquatic Feed Co.	Grouper feed 8925	133
Hainan Haiyi Aquatic Feed Co.	Grouper feed 8925	213
Shang Shang Biotechnology Co.	Grouper Fry feed 2L#	239
Shang Shang Biotechnology Co.	Grouper juvenile feed 4#	282
Shang Shang Biotechnology Co.	Grouper medium-sized fish feed 6#	121
Shang Shang Biotechnology Co.	Grouper adult feed 8#	118
Zhanjiang Haida Feed Co.	Grouper feed 731 (No. 10 enlarged)	96
Guangdong Haida Feed Co.	Grouper feed 511	59
Shuanghu Food Co.	Grouper feed No. 8	90
Fujian Tianma Feed Co.	Grouper feed No. 7	171

Zhanjiang Aohua Aquatic Feed Co.	Grouper feed No. 8	541
Zhanjiang Yuehai Feed Co.	Grouper feed No. 9	256
Zhanjiang Haida Feed Co.	Grouper compound feed 602	185
Zhanjiang Haida Feed Co.	Grouper compound feed 604	379
Zhanjiang Haida Feed Co.	Grouper compound feed 605	285
Zhanjiang Haida Feed Co.	Grouper compound feed 606	173
Zhanjiang Haida Feed Co.	Grouper compound feed 607	185
Zhanjiang Haida Feed Co.	Grouper compound feed 608	207
Zhanjiang Haida Feed Co.	Grouper compound feed 609	160
Zhanjiang Haida Feed Co.	Grouper compound feed 610	151
Zhanjiang Haida Feed Co.	Grouper compound feed 611	171
Zhanjiang Haida Feed Co.	Grouper compound feed 612	176
Zhanjiang Haida Feed Co.	Grouper compound feed 613	117
Zhanjiang Haida Feed Co.	Grouper compound feed 615	112
Zhanjiang Guolian Feed Co.	Grouper compound feed 4#	222
Zhanjiang Guolian Feed Co.	Grouper compound feed 5#	270
Zhanjiang Guolian Feed Co.	Grouper compound feed 6#	172
Zhanjiang Guolian Feed Co.	Grouper compound feed 7#	337
Zhanjiang Guolian Feed Co.	Grouper compound feed 8#	230
Zhanjiang Guolian Feed Co.	Grouper compound feed 9#	262
Zhanjiang Guolian Feed Co.	Grouper compound feed 10#	307
Zhanjiang Guolian Feed Co.	Grouper compound feed 11#	292
Guangdong Yuejia Feed Co.	Grouper adult feed No. 9 9679	442

Fujian Tianma Feed Co.	Grouper feed No. 9	862
Fujian Tianma Feed Co.	Grouper feed No. 8	348
Guangdong Haida Feed Co.	Grouper feed No. 9 609A	328
Zhongshan Unity Feed Co.	Grouper feed No. 4	404
Zhongshan Unity Feed Co.	Grouper feed No. 5	321
Fujian Yuehai Feed Co.	Grouper feed No. 5	142
Yangjiang Dahai Aquatic Feed Co.	Grouper medium-sized fish feed No. 5	116
Yangjiang Dahai Aquatic Feed Co.	Grouper medium-sized fish feed No. 6	276
Yangjiang Dahai Aquatic Feed Co.	Grouper medium-sized fish feed No. 7 B	166
Yangjiang Dahai Aquatic Feed Co.	Grouper medium-sized fish feed No. 8 B	123
Yangjiang Dahai Aquatic Feed Co.	Grouper medium-sized fish feed No. 9 B	402
Yangjiang Dahai Aquatic Feed Co.	Grouper medium-sized fish feed No. 10 B	84.1
Yangjiang Dahai Aquatic Feed Co.	Grouper medium-sized fish feed No. 11 B	247
Yangjiang Dahai Aquatic Feed Co.	Grouper medium-sized fish feed No. 12 B	301
Yangjiang Base	Grouper feed 5B	136
Yangjiang Base	Grouper feed 6B	209
Yangjiang Base	Grouper feed 7B	118
Yangjiang Base	Grouper feed 8B	131
Yangjiang Base	Grouper feed 9B	151
Yangjiang Base	Grouper feed 10B	179
-	Grouper compound feed 4#	408
Zhangzhou Hai Rui Aquatic Biotechnology Co.	Grouper compound feed 5#	166
Fujian Tianma Feed Co.	Grouper compound feed 5#	484

Zhongshan Unity Feed Co.	Grouper compound feed 5#	316
Zhongshan Unity Feed Co.	EP 503 Grouper compound feed 3#	522
Zhongshan Unity Feed Co.	EP 504 Grouper compound feed 4#	389
Zhongshan Unity Feed Co.	EP 506 Grouper compound feed 5#	417
Yangjiang Dahai Aquatic Feed Co.	Grouper medium-sized fish feed No. 7 B	111
Yangjiang Dahai Aquatic Feed Co.	Grouper adult feed No.10 B	133
Fujian Tianma Feed Co.	Grouper pellet feed No. 5	481
Haixing Haibao Feed Co.	Grouper feed No. 4	279
Hainan Hengxing Feed Industry Co.	Grouper medium-sized fish compound feed No. 5	160
Yangjiang Base	Grouper medium-sized fish feed No. 5	172
Yangjiang Base	Grouper medium-sized fish feed No. 5 B	158
Yangjiang Base	Grouper medium-sized fish feed No. 6 B	161
Yangjiang Base	Grouper medium-sized fish feed No. 7 B	144
Yangjiang Base	Grouper adult feed No.8 B	139
Yangjiang Base	Grouper adult feed No.9 B	183
Zhangzhou Hai Rui Aquatic Biotechnology Co.	Grouper adult feed No.7	272
Zhangzhou Hai Rui Aquatic Biotechnology Co.	Grouper adult feed No.11	176
Hainan Company	Grouper adult feed No.8	162
Hainan Company	Grouper medium-sized fish feed No. 5	294
Hainan Company	Grouper adult feed No.9	284
Hainan Company	Grouper medium-sized fish feed No. 6 B	106
Hainan Company	Grouper medium-sized fish feed No. 7 B	96
Hainan Company	Grouper adult feed No.8 B	232

Hainan Company	Grouper adult feed No.10 B	173
Hainan Company	Grouper adult feed No.13 B	115
Fuxing (Xiamen) Bio-Feed Co.	Plectropomus leopardus feed No. 6	83
Fuxing (Xiamen) Bio-Feed Co.	Plectropomus leopardus feed No. 8	156
Fuxing (Xiamen) Bio-Feed Co.	Plectropomus leopardus feed No. 10	144
Nippon Hayashiken Sangyo Co.	Plectropomus leopardus feed No. 3	326
Nippon Hayashiken Sangyo Co.	Plectropomus leopardus feed No. 4	334
Nippon Hayashiken Sangyo Co.	Plectropomus leopardus feed No. 7	212
Hainan Company	Plectropomus leopardus feed No. 8	122
Hainan Company	Plectropomus leopardus feed No. 8	84
Yangjiang Base	Grouper feed 7B	107
Yangjiang Base	Grouper feed 8B	105
Yangjiang Base	Grouper feed 9B	137
Yangjiang Base	Grouper feed 10B	69
Yangjiang Base	Grouper feed 11B	65
Yangjiang Base	Grouper feed 12B	86
Shang Shang Biotechnology Co.	Grouper feed No. 6	1050
Shang Shang Biotechnology Co.	Grouper feed No. 7	191
Shang Shang Biotechnology Co.	Grouper feed No. 9	357
Shang Shang Biotechnology Co.	Grouper feed No. 10	246
Zhanjiang Haida Feed Co.	Grouper feed No. 5	286
Zhanjiang Haida Feed Co.	Grouper feed No. 8	830
Hainan Haiyi Aquatic Feed Co.	Grouper feed No. 5	205

Hainan Haiyi Aquatic Feed Co.	Grouper feed No. 7	255
Foshan Jie Da Feed Co.	Grouper feed No. 6	211
Zhanjiang Yuehai Feed Co.	Grouper feed No. 7	666
Guangdong Yuejia Feed Co.	Grouper feed No. 3	552
Santong Central Feed (Shandong) Co.	Plectropomus leopardus feed No. 10	335
Nippon Hayashiken Sangyo Co.	Plectropomus leopardus feed No. 7	463
Nippon Hayashiken Sangyo Co.	Plectropomus leopardus feed No. 8	188
Zhanjiang Haida Feed Co.	Plectropomus leopardus feed No. 10	126
Zhanjiang Haida Feed Co.	Plectropomus leopardus feed 810	150
Santong Central Feed (Shandong) Co.	Plectropomus leopardus feed No. 10	158
Foshan Jie Da Feed Co.	Plectropomus leopardus feed No. 6	806
Shandong Hanye Biotechnology Co.	Plectropomus leopardus feed No. 6 EP	310
Nisshin Marubeni Feed Co.	Plectropomus leopardus feed No. 5	243
Nisshin Marubeni Feed Co.	Plectropomus leopardus feed No. 10	136
Fuxing (Xiamen) Bio-Feed Co.	Plectropomus leopardus adult feed No. 6	398
Fuxing (Xiamen) Bio-Feed Co.	Plectropomus leopardus adult feed No. 8	1120
Yangjiang Haihai Aquatic Feed Co.	Plectropomus leopardus medium-sized fish feed No. 6 B	27
